# Cost-effectiveness analysis of triplet therapy of encorafenib, binimetinib, and cetuximab for treatment of metastatic BRAF V600E-mutated colorectal cancer in China

**DOI:** 10.3389/fphar.2026.1746058

**Published:** 2026-08-10

**Authors:** Mengmeng Liu, Tong Liu

**Affiliations:** Department of Pharmacy, Harbin Medical University Cancer Hospital, Harbin, China

**Keywords:** binimetinib, cetuximab, colorectal cancer, cost-effectiveness, encorafenib, pharmacoeconomics

## Abstract

**Background:**

This study evaluates the cost-effectiveness of triplet therapy of encorafenib, binimetinib, and cetuximab in contrast to doublet therapy of encorafenib and cetuximab or a control group of cetuximab plus FOLFIRI as second-line or subsequent treatment for advanced or metastatic colorectal cancer (mCRC) patients with BRAF V600 E mutation-positive status in China.

**Methods:**

An economic evaluation using a 3-state partitioned survival model assessed the cost-effectiveness of triplet therapy versus doublet therapy, triplet therapy versus the control group, or doublet therapy versus the control group.

**Results:**

The anticipated costs for triplet therapy, doublet therapy and control therapy were 58,614.81 USD, 46,521.37 USD and 15,710.17 USD, respectively. The estimated utilities of triplet therapy, doublet therapy and control therapy were 0.84 QALYs, 0.76 QALYs and 0.53 QALYs. The ICER of triplet-therapy vs. doublet-therapy or triplet-therapy vs. control therapy or doublet-therapy vs. control therapy was 154,675.20 USD/QALY, 140,355.45 USD/QALY, or 135,434.11 USD/QALY.

**Conclusion:**

Our results suggested that triplet therapy was not cost-effective compared to doublet therapy and control therapy as second-line or subsequent treatment for advanced or mCRC patients with BRAF V600 E mutation at a WTP threshold of 40,000 USD/QALY.

## 1 Introduction

Colorectal cancer (CRC) is the second most common cause of cancer death in the United States (United States of America). In 2023, approximately 153,020 individuals were diagnosed with CRC, and 52,550 died from the disease, including 19,550 cases and 3,750 deaths in individuals younger than 50 years (Siegel et al., 2023). CRC threatens health and causes severe social burdens, accounting for about 10% of all new cancer cases (Sung et al., 2021). Data from the National Cancer Center of China in 2022 show that CRC ranks fourth in incidence and fifth in mortality among male patients, third in incidence and fourth in mortality among female patients (Xia et al., 2022). In 2022, the incidence of patients newly diagnosed with CRC in China was 517,100, and the estimated number of deaths from CRC was 240,000, indicating that CRC poses a huge burden in China (Zheng et al., 2024). Mutations in BRAF are recurrently detected in human cancer, including melanoma, colorectal, thyroid, non-small-cell lung, and hairy-cell leukemia (Davies et al., 2002). BRAF encodes a serine/threonine protein kinase that is part of the RAS/RAF/MEK/ERK pathway. The majority of mutations in BRAF result in V600 E substitution, and these patients generally have a poor prognosis. Approximately 10% of patients with metastatic colorectal cancer (mCRC) have a BRAF mutation, with recent estimates ranging from as low as 5% to as high as 21%. BRAF V600 E mutation results in downstream phosphorylation of MEK and ERK, leading to constitutive activation of the mitogen-activated protein kinase (MAPK) signaling pathway, which drives tumor cell proliferation and survival (Tabernero et al., 2021).

According to the National Comprehensive Cancer Network (NCCN, 2025) guideline (Colon Cancer, version 3, 2025), the therapy regimen for advanced or mCRC patients with BRAF V600 E mutation was Encorafenib+(cetuximab or panitumumab) or Encorafenib+(cetuximab or panitumumab)+FOLFOX (NCCN, 2025). Encorafenib is a highly selective, ATP-competitive small-molecule BRAF inhibitor with anti-proliferative and apoptotic activity in tumor cells expressing BRAF V600 E and has prolonged pharmacodynamic activity compared with other approved BRAF inhibitors (Delord et al., 2017). BRAF V600 E inhibition causes rapid pathway feedback reactivation through the epidermal growth factor receptor (EGFR) (Corcoran et al., 2015). Previous clinical trials targeting BRAF simultaneously with EGFR inhibition have shown the value of this combination in targeting MAPK signaling (Kopetz et al., 2025). Cetuximab, a chimerized monoclonal antibody to EGFR, specifically bound to the EGFR with high affinity by occluding the ligand-binding region, blocking ligand-induced EGFR tyrosine kinase activation and subsequent signal-transduction events leading to cancer cell proliferation (Yao et al., 2025). There is an open-label, phase three trial reporting the overall survival and safety of the triplet therapy arm (encorafenib, binimetinib, and cetuximab), doublet therapy arm (encorafenib and cetuximab), and control arm (cetuximab and irinotecan or cetuximab and FOLFIRI) (Kopetz et al., 2019).

Binimetinib (also known as MEK162 or ARRY-438162) is a potent and selective allosteric, ATP-uncompetitive inhibitor of MEK1 and MEK2 (Bendell et al., 2017). In most cancers, the ERK pathway, including RAS, BRAF, CRAF, and MEK1 or MEK2, is hyperactive due to deregulation of receptor tyrosine kinases. MEK1 and MEK2 are uniquely positioned within the ERK pathway, where they process inputs from multiple upstream activating kinases following RAF activation, making them attractive drug targets. Binimetinib has been investigated both as a single agent and in combination with other agents in patients with selected advanced or mCRC (Elez et al., 2024). In our study, we will conduct a cost-effectiveness analysis of encorafenib, binimetinib, and cetuximab compared to encorafenib and cetuximab or control therapy as the second-line and subsequent treatment for advanced or mCRC patients with BRAF V600 E mutation in China. As few studies are reporting the cost-effectiveness of binimetinib-based therapy for the treatment of CRC, there is only one study evaluating the economic impact of binimetinib-based therapy in the United States of America, and several studies have conducted the cost-effectiveness analysis for the treatment of melanoma (Li et al., 2021; Trouiller et al., 2024; Halloush et al., 2023; Blommestein et al., 2025). Therefore, it is important to apply cost-effectiveness analysis to conduct an economic evaluation of encorafenib, binimetinib, and cetuximab in the treatment of advanced or mCRC patients with BRAF V600 E mutation in China.

## 2 Methods and materials

### 2.1 Target population and treatment strategies

Our study adhered to the provisions of the Consolidated Health Economic Evaluation Reporting Standards (CHEERS) (Husereau et al., 2022). The source of efficacy and safety data of the triplet therapy arm, doublet therapy arm, and control arm was from a global, multi-center, randomized, open-label, phase three trial (BEACON) (Kopetz et al., 2019). The clinical trial randomly assigned patients in a 1:1:1 ratio to one of three groups. Randomization was stratified according to Eastern Cooperative Oncology Group (ECOG) performance status score (0 or 1), previous use of irinotecan (yes or no), and cetuximab formulation. Since there were no significant differences in baseline characteristics among the groups, it was considered that the results between the groups could be compared. Patients in the triplet therapy group received encorafenib (300 mg daily), binimetinib (45 mg twice daily), and cetuximab (400 mg per square meter of body-surface area as an initial dose, then 250 mg per square meter weekly). Patients in the doublet therapy group received encorafenib and cetuximab, administered in the same doses and on the same schedule as the triplet regimen. Patients in the control group received the investigators’ choice of either cetuximab (administered in the same doses and on the same schedule as the other regimens) and irinotecan (180 mg per square meter on days 1 and 15) or cetuximab and FOLFIRI (folinic acid [180 mg per square meter, administered on days 1 and 15], fluorouracil [400 mg per square meter as an initial dose, then 1,200 mg per square meter per day for 2 days, initiated on days 1 and 15], and irinotecan [at the same dose and on the same schedule as the other regimens]). Treatment was administered until disease progression, unacceptable toxic effects, withdrawal of consent, initiation of subsequent anticancer therapy, or death (Kopetz et al., 2019). The cost-effectiveness was assessed by calculating the incremental cost-effectiveness ratio (ICER), which measures the ratio between cost increments and quality-adjusted life years (QALYs) increments.

### 2.2 Disease modeling

**FIGURE 1:**
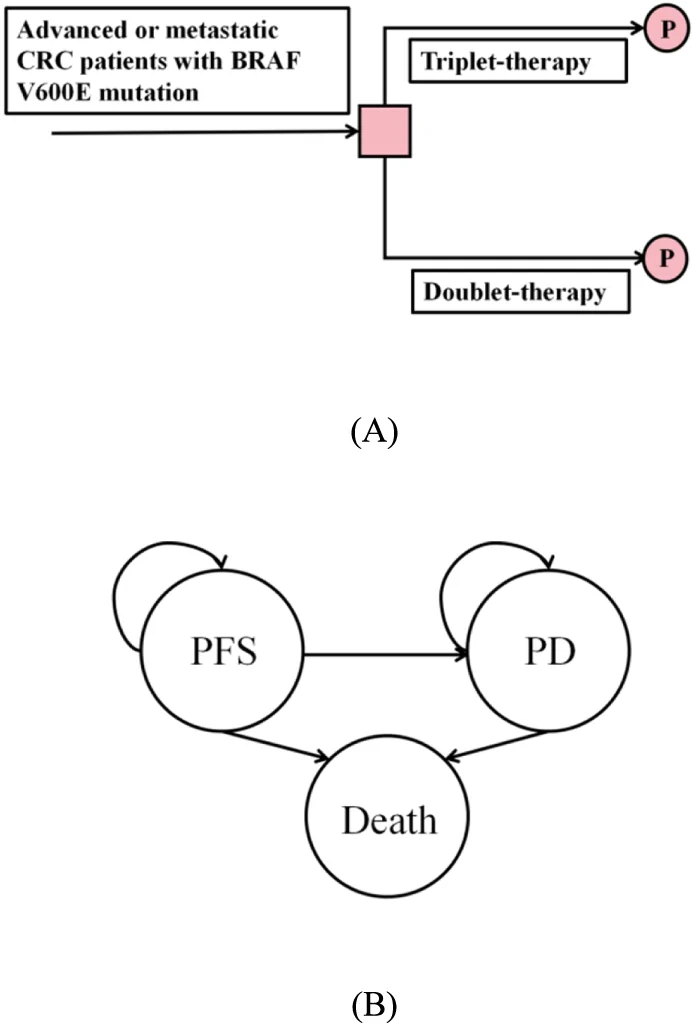
Model structure of a decision tree and a diagram of the partitioned survival model structure **(A)** A decision tree **(B)** Partitioned survival model structure with 3 health states. Abbreviation: CRC: colorectal cancer; PFS: progression-free survival; PD: progressive disease.

**FIGURE 2:**
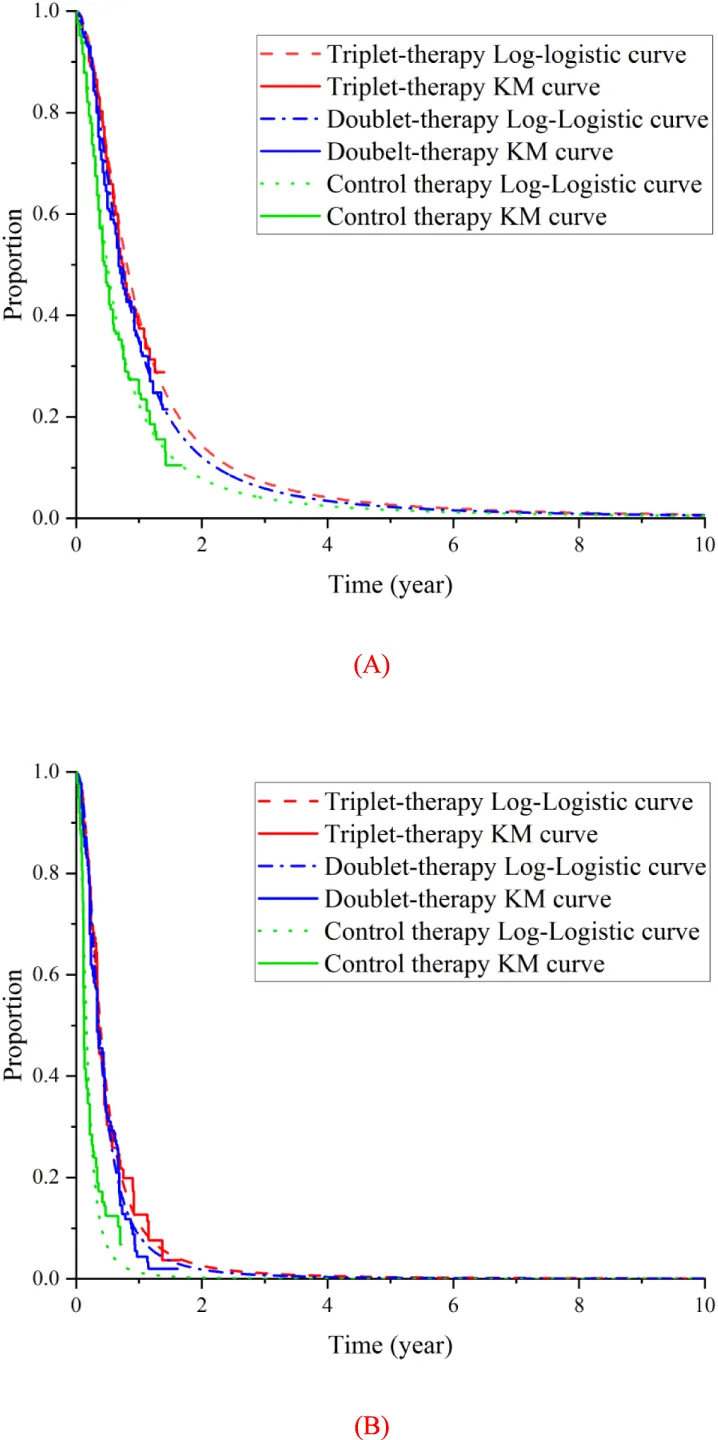
Kaplan-Meier and Log-logistic model curves to assess the plausibility of long-term extrapolations **(A)** OS curve in the triplet-therapy arm, doublet-therapy arm, and control therapy **(B)** PFS curve in the triplet-therapy arm, doublet-therapy arm, and control therapy. Abbreviation: KM: Kaplan-Meier.

A partitioned survival model (PSM) was employed for this analysis. According to the official website of the National Health Commission of China, the standard height and weight of the Chinese population are 167.1 cm and 66.2 kg (body surface area of 1.73 m^2^), respectively (National Health Commission, 2020). A PSM was a commonly used model in the economic evaluation of oncology drugs. The PSM used the area under the curve to represent the number of patients in each state. It was mainly used to evaluate the impact of interventions that could prolong the life of the patient on the expected lifetime and quality of life of the patient (Rui et al., 2021). Three health states were used by TreeAge Pro Healthcare 2022 software to frame the PSM in our study, including progression-free survival (PFS), progressed disease (PD), and death, as illustrated in Figure 1. Software Getdata 2.25 was applied to extract and digitize the parametric survival curves reported in the study to simulate long-term survival of advanced or mCRC patients with BRAF V600 E mutation. Seven distinct survival models were employed for the survival extrapolation from PFS and OS curves that had already been reported, such as Exponential, Gamma, Gompertz, Weibull (AFT), Weibull (PH), Log-Logistic, and Log-Normal models. Specifically, deducing the potential survival distribution from a Kaplan-Meier graph was advantageous for determining the parameters of these models (Hoyle and Henley, 2011). Selecting the most suitable survival model was based on the evaluation of all the fitted curves through a visualized approach, the lowest Akaike information criterion value (AIC), and the lowest Bayesian information criterion value (BIC) (Table 1). The Log-Logistic distribution was chosen due to the AIC values, BIC values, and its clinical plausibility compared to the other models. The fitted model curves selected the 10-year time horizon to indicate the entire life cycle of a patient. This assumption could be deduced from the model prediction that more than 99.3% of patients in the triplet therapy arm had deceased by this time. To explore the structural uncertainty related to long-term extrapolation, we applied the Gamma model, Log-Normal model, and Weibull model, which had lower AIC values and BIC values to simulate the survival of mCRC patients (Figure 2).

**TABLE 1:** Akaike information criterion value and Bayesian information criterion value in different models.

Survival	Models	Triplet-therapy		Doublet-therapy		Control therapy	
		AIC	BIC	AIC	BIC	AIC	BIC
OS	Exponential	658.05	661.46	666.39	669.78	711.09	714.49
	Gamma	634.25	641.07	643.11	649.89	697.21	704.01
	Gompertz	649.85	656.67	658.35	665.14	709.99	716.78
	Weibull (AFT)	637.07	643.90	645.90	652.69	700.29	707.09
	Weibull (PH)	637.07	643.90	645.90	652.69	700.29	707.09
	Log-logistic	631.85	638.68	640.54	647.33	692.93	699.73
	Log-normal	633.17	639.99	643.97	650.76	695.59	702.39
PFS	Exponential	691.78	695.20	760.88	764.28	576.38	579.78
	Gamma	655.23	662.06	712.36	719.15	543.03	549.82
	Gompertz	683.56	690.38	742.46	749.25	578.04	584.83
	Weibull (AFT)	662.48	669.31	718.87	725.66	558.40	565.19
	Weibull (PH)	662.48	669.31	718.87	725.66	558.40	565.19
	Log-logistic	649.08	655.90	713.00	719.79	507.36	514.16
	Log-normal	650.51	657.33	713.78	720.57	510.88	517.68

Abbreviation: OS, overall survival; PFS, progression-free survival.

### 2.3 Costs and utility

From a payer perspective, this model exclusively included the direct expenditure for medical care services, such as drug fees, costs of BSC (Best Supportive Care), management fees for serious adverse events (SAEs), and follow-up costs (imaging examination and laboratory test fees, etc.). As Binimetinib was not on the market in China, in order to obtain the lowest price of the drug, we chose the Lao generic drug of Binimetinib, and its price was provided by the overseas drug purchase platform website (https://www.zhaoyaow.com/bmtn/jg/14380.html), through which patients could obtain overseas drugs (Captain Medicine, 2026) (Binimetinib: 15mg × 180 tablets; 5420 CYN per box; 1 CYN = 0.1472 USD). The costs of Encorafenib, Cetuximab, Irinotecan, Folinic acid, and Fluorouracil were from the big data service platform for China’s health industry (Yao, 2025). The costs of follow-up care, BSC, and SAEs management were derived from the published literature (Huang et al., 2024; Li et al., 2021). In our study, adverse drug reactions with an incidence rate exceeding 5% and a grade surpassing or equal to grade 3 were selected to calculate the costs of SAEs management for the triplet therapy arm and doublet therapy arm. Among them, the costs of SAEs involved diarrhea, nausea, abdominal pain, asthenia, and hemoglobin increased (Kopetz et al., 2019). The health utility index referred to an aggregate indicator of physical health with weights varying from different states (Drummond et al., 1997). A range from 0 to 1 (0 denotes death and one denotes perfect health) was commonly used for the utility scale. Given the absence of quality-of-life statistics from the clinical trial in this study, the utility values for the PFS and PD of advanced or mCRC were sourced from the previously published literature [ (Huang et al., 2024)]. The yearly discount rate in our study was 5% (Liu et al., 2020) (Tables 1 and 2).

**TABLE 2:** Cost inputs in the PSM.

Items	Date (Year)	Country	Dose	Therapeutic schedule	CPI	Cost in 2024 per cycle (USD)	Low	High	Distribution	References
Binimetinib	2026	Laos	45 mg	45 mg twice daily, every 14 days	—	372.51	298.01	447.01	Gamma	23
Encorafenib	2026	China	300 mg	300 mg orally once daily, every 14 days	—	1715.46	1,372.37	2058.55	Gamma	24
Cetuximab	2025	China	500 mg/cm2; BSA: 1.73 cm^2^	500 mg/cm2, every 14 days	—	1743.75	1,395.00	2092.50	Gamma	24
Irinotecan	2025	China	180 mg/m2; BSA: 1.73 cm^2^	180 mg/m2 IV over 30–90 min, day 1	—	63.21	50.57	75.85	Gamma	24
Folinic acid	2025	China	400 mg/m2; BSA: 1.73 cm^2^	400 mg/m2 IV infusion to match duration of irinotecan infusion, day 1	—	24.27	19.41	29.12	Gamma	24
Fluorouracil	2025	China	400 mg/m2; 1,200 mg/m2; BSA: 1.73 cm^2^	400 mg/m2 IV bolus day 1, followed by 1,200 mg/m2 /day ×2 days continuous infusion	—	7.03	5.62	8.43	Gamma	24
BSC	2024	China	—	—	—	385.71	308.57	462.85	Gamma	25
Follow-up	2024	China	—	—	—	271.51	217.21	325.81	Gamma	25
Diarrhea	2021	United States	—	—	2021: 0.9% 2022: 2% 2023: 0.2% 2024: 0.2%	10,739.57	8591.66	12,887.48	Gamma	15
Nausea	2021	United States	—	—		10,739.57	8591.66	12,887.48	Gamma	15
Abdominal pain	2021	United States	—	—		10,739.57	8591.66	12,887.48	Gamma	15
Asthenia	2021	United States	—	—		11,054.99	8843.99	13,265.99	Gamma	15
Hemoglobin increased	2021	United States	—	—		12,128.23	9702.58	14,553.87	Gamma	15

Abbreviation: CPI, consumer price index; USD, united states dollar.

### 2.4 Sensitivity analysis

In this study, both one-way sensitivity analysis and probabilistic sensitivity analysis (PSA) were employed for uncertainty measurement and robustness evaluation of the model. Among them, the cost parameters obtained from the one-way sensitivity analysis ranged within ±20% of their baseline values, while the utility parameters ranged within ±10% of their baseline values (Tables 2 and 3). A Monte Carlo simulation was performed 1,000 times for PSA, with each iteration randomly sampling from the distributions of all parameters. To illustrate the uncertainty of potential willingness-to-pay (WTP) thresholds, the cost-effectiveness acceptability curves were employed (Aguiar-Ibáñez et al., 2022). The WTP threshold in the cost-effectiveness acceptability curves was set at 40,000 USD/QALY, which was three times the *per capita* GDP of China in 2024 from the National Bureau of Statistics (Liu et al., 2020). In the cost-effectiveness analysis, the decision-making was based on an incremental analysis. An incremental analysis was the comparison of costs and outcomes between the intervention and the comparator. If the intervention had the lowest cost and better outcome compared to the comparator, it would be the strictly dominant regimen. In contrast, if the intervention had a higher cost and a worse outcome compared to the comparator, it would be the strictly dominated regimen. If the intervention regimen had both a higher cost and a better outcome compared to the comparator, the ICER, which was the ratio of the difference in costs to the difference in outcomes between the two regimens, needed to be calculated. If the ICER was smaller than or equal to the threshold value, the intervention was more cost-effective than the comparator; if the ICER was larger than the threshold value, the intervention was not cost-effective compared to the comparator. In incremental analysis, the WTP threshold value for QALY was recommended to be 1–3 times the GDP *per capita* (Liu et al., 2020). We initially adopted 3 times the GDP *per capita* as the WTP threshold. If the ICER was greater than 3 times the GDP *per capita*, it indicated that the result lacked economic advantage. Even if the WTP threshold was set at one time the GDP *per capita*, the result would be even less cost-effective. The WTP threshold was defined by the WHO as a value representing an estimate of what a consumer of healthcare might be prepared to pay for the health benefit. These thresholds were often based on cost-effectiveness ratios estimating health gains for the resources expended (Pron et al., 2023). The cost parameters were modeled using a gamma distribution, whereas the utility value parameters and SAE rate parameters were modeled with a beta distribution (Courtney et al., 2021).

**TABLE 3:** Other inputs in the PSM.

Items	Value	Low	High	Distribution	References
Utility (QALYs)					
PFS	0.84	0.76	0.92	Beta	27
PD	0.57	0.51	0.63	Beta	27
Other					
BSA	1.73	1.38	2.08	Normal	20
Discounting rate	0.05	0	0.08	Fixed	28
SAE rate in the triplet-therapy arm					
Diarrhea	0.10	0.08	0.12	Beta	12
Nausea	0.05	0.04	0.06	Beta	12
Abdominal pain	0.06	0.05	0.07	Beta	12
Hemoglobin increased	0.11	0.09	0.13	Beta	12
SAE rate in the doublet-therapy arm					
Diarrhea	0.10	0.08	0.12	Beta	12
Abdominal pain	0.05	0.04	0.06	Beta	12
Asthenia	0.05	0.04	0.06	Beta	12

Abbreviation: PFS, progression-free survival; PD, progressive disease; QALYs, quality-adjusted life years; BSA, body surface area; SAE, serious adverse events.

## 3 Results

### 3.1 Base case findings

As depicted in Table 4, the anticipated costs for triplet therapy, doublet therapy and control therapy were 58,614.81 USD, 46,521.37 USD and 15,710.17 USD, respectively. The estimated utilities of triplet therapy, doublet therapy and control therapy were 0.84 QALYs, 0.76 QALYs and 0.53 QALYs. The ICER of triplet therapy vs. doublet therapy or triplet therapy vs. control therapy or doublet therapy vs. control therapy was 154,675.20 USD/QALY, 140,355.45 USD/QALY, or 135,434.11 USD/QALY. Our result suggested that compared with the control therapy and doublet therapy, triplet therapy was not cost-effective as second-line or subsequent treatment for advanced or mCRC patients with BRAF V600 E mutation at a WTP threshold of 40,000 USD/QALY.

**TABLE 4:** Economic evaluation results.

Different scenario	Cost (USD)	Utility (QALYs)	IC (USD)	I.E., (QALYs)	ICER (USD/QALYs)
The time horizon was 10 years					
Comparison 1					
Triplet regimen	58,614.81	0.84	12,093.44	0.08	154,675.20
Doublet regimen	46,521.37	0.76	—	—	—
Comparison 2					
Triplet regimen	58,614.81	0.84	42,904.64	0.31	140,355.45
Cetuximab + FOLFIRI	15,710.17	0.53	—	—	—
Comparison 3					
Doublet regimen	46,521.37	0.76	30,811.20	0.23	135,434.11
Cetuximab + FOLFIRI	15,710.17	0.53	—	—	—
The time horizon was 5 years					
Comparison 1					
Triplet regimen	58,540.64	0.80	12,080.76	0.07	171,007.10
Doublet regimen	46,459.88	0.73	—	—	—
Comparison 2					
Triplet regimen	58,540.64	0.80	42,878.06	0.29	148,033.49
Cetuximab + FOLFIRI	15,662.58	0.51	—	—	—
Comparison 3					
Doublet regimen	46,459.88	0.73	30,797.30	0.22	140,622.91
Cetuximab + FOLFIRI	15,662.58	0.51	—	—	—
The gamma model was applied					
Comparison 1					
Triplet regimen	55,848.68	0.69	11,729.53	0.06	197,313.76
Doublet regimen	44,119.15	0.63	—	—	—
Comparison 2					
Triplet regimen	55,848.68	0.69	26,003.00	0.19	139,149.65
Cetuximab + FOLFIRI	29,845.69	0.51	—	—	—
Comparison 3					
Doublet regimen	44,119.15	0.63	14,273.47	0.13	112,014.96
Cetuximab + FOLFIRI	29,845.69	0.51	—	—	—
The log-normal model was applied					
Comparison 1					
Triplet regimen	59,459.55	0.86	13,224.37	0.08	168,885.23
Doublet regimen	46,235.18	0.78	—	—	—
Comparison 2					
Triplet regimen	59,459.55	0.86	43,019.47	0.32	134,381.44
Cetuximab + FOLFIRI	16,440.07	0.54	—	—	—
Comparison 3					
Doublet regimen	46,235.18	0.78	29,795.11	0.24	123,209.01
Cetuximab + FOLFIRI	16,440.07	0.54	—	—	—
The weibull model was applied					
Comparison 1					
Triplet regimen	56,132.96	0.68	11,698.91	0.06	211,995.16
Doublet regimen	44,434.06	0.62	—	—	—
Comparison 2					
Triplet regimen	56,132.96	0.68	26,103.65	0.17	151,752.60
Cetuximab + FOLFIRI	30,029.31	0.51	—	—	—
Comparison 3					
Doublet regimen	44,434.06	0.62	14,404.74	0.12	123,296.89
Cetuximab + FOLFIRI	30,029.31	0.51	—	—	—
Volume-based procurement + Beijing medical insurance					
Comparison 1					
Triplet regimen	12,893.81	0.84	4,581.47	0.08	58,597.08
Doublet regimen	8,312.34	0.76	—	—	—
Comparison 2					
Triplet regimen	12,893.81	0.84	6,470.90	0.31	21,168.47
Cetuximab + FOLFIRI	6,422.91	0.53	—	—	—
Comparison 3					
Doublet regimen	8,312.34	0.76	1889.43	0.23	8,214.91
Cetuximab + FOLFIRI	6,422.91	0.53	—	—	—
Volume-based procurement + heilongjiang province medical insurance					
Comparison 1					
Triplet regimen	14,943.20	0.84	4,918.19	0.08	62,903.67
Doublet regimen	10,025.01	0.76	—	—	—
Comparison 2					
Triplet regimen	14,943.20	0.84	8,104.00	0.31	26,510.90
Cetuximab + FOLFIRI	6,839.20	0.53	—	—	—
Comparison 3					
Doublet regimen	10,025.01	0.76	3,185.81	0.23	13,851.35
Cetuximab + FOLFIRI	6,839.20	0.53	—	—	—

Abbreviation: USD, united states dollar; QALYs, quality-adjusted life years; IC, incremental cost; I.E.,, incremental effectiveness; ICER, incremental cost-effectiveness ratio; FOLFIRI, Fluorouracil + Folinic acid + Irinotecan.

### 3.2 Sensitivity analysis

**FIGURE 3:**
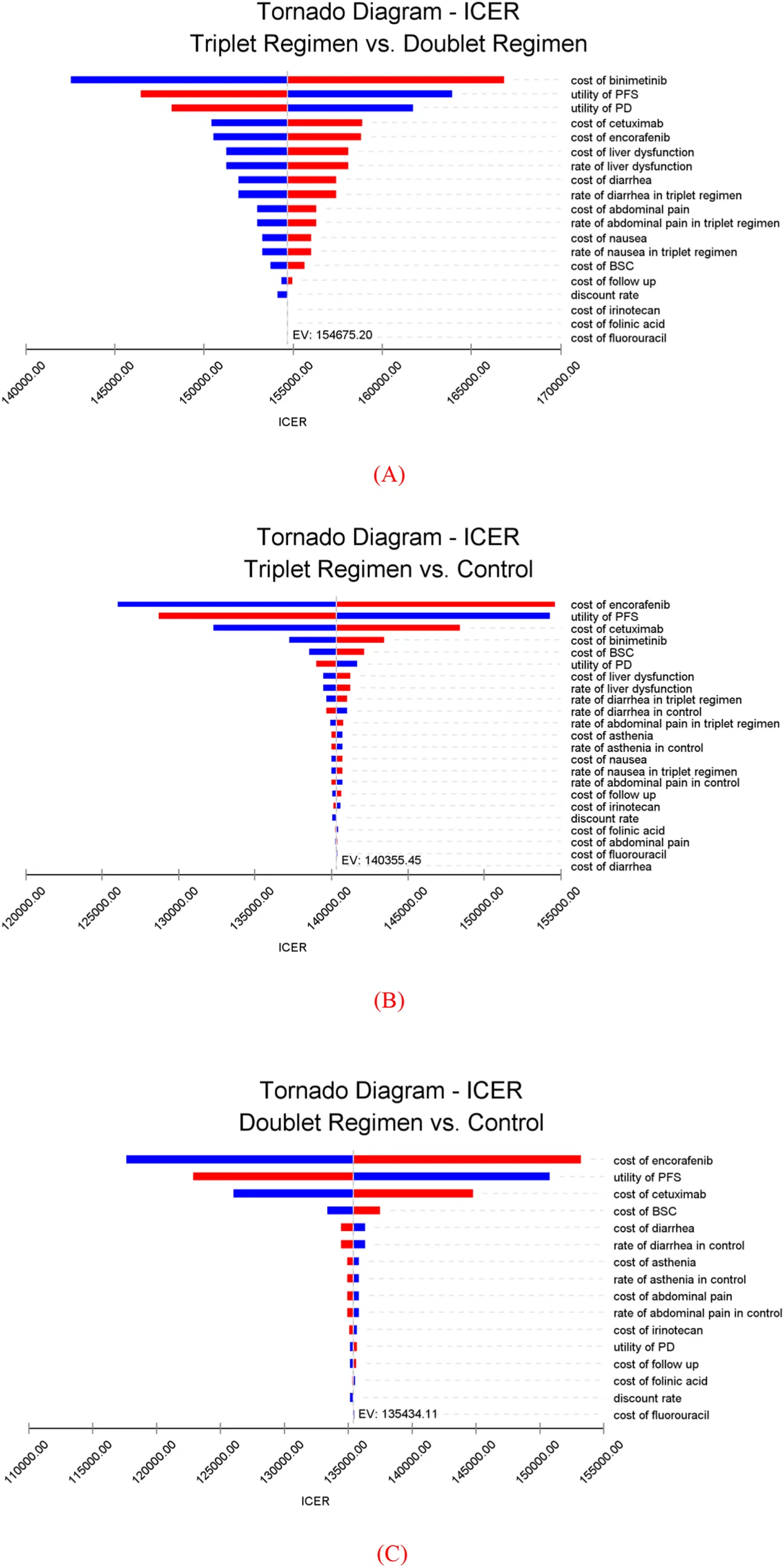
The tornado diagrams showed the outcome of the one-way sensitivity analysis **(A)** Triplet-therapy vs. Doublet-therapy **(B)** Triplet-therapy vs. Control group **(C)** Doublet-therapy vs. Control group. Abbreviation: ICER: incremental cost-effectiveness ratio.

**FIGURE 4:**
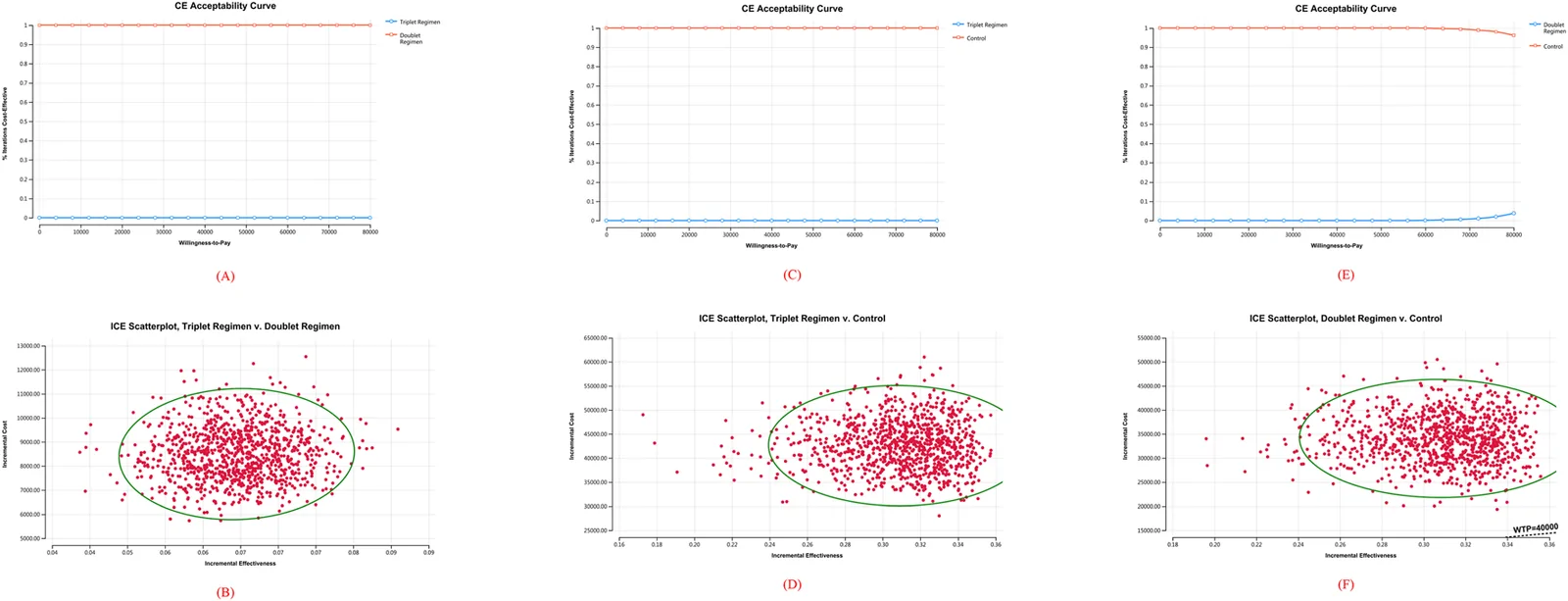
Probabilistic sensitivity analysis revealed that triplet-therapy was not cost-effective compared to doublet-therapy or the control group at the WTP threshold, and doublet-therapy was not cost-effective compared to the control group **(A,B)** Triplet-therapy vs. Doublet-therapy **(C,D)** Triplet-therapy vs. Control group **(E,F)** Doublet-therapy vs. Control group.

Tornado diagrams were employed to visualize the outcomes of the one-way sensitivity analysis (Figure 3). Our results revealed that the most influential factor on the ICER of triplet therapy vs. doublet therapy was the cost of binimetinib, the most influential factor on the ICER of triplet therapy vs. cetuximab plus FOLFIRI treatment was the cost of encorafenib, and the most influential factor on the ICER of doublet therapy vs. cetuximab plus FOLFIRI treatment was the cost of encorafenib. The PSA revealed a 100% possibility that triplet therapy was not cost-effective compared to doublet therapy or cetuximab plus FOLFIRI treatment with a 10-year time horizon at the threshold of 40,000 USD/QALY. The PSA also indicated a 100% possibility that doublet therapy was not cost-effective compared to cetuximab plus FOLFIRI treatment with a 10-year time horizon at the threshold of 40,000 USD/QALY (Figure 4).

### 3.3 Scenario analysis

To explore the uncertainty of models applied to simulate the survival of the mCRC patients with BRAF V600 E mutation. We employed the Gamma model, Log-Normal model, and Weibull model, which had the lower AIC or BIC values, to complete the long-term extrapolation as the scenario analysis. The scenario results suggested that no matter which model was applied, triplet therapy was still not cost-effective compared with the control therapy or doublet therapy as second-line or subsequent treatment for mCRC patients with a BRAF V600 E mutation at a WTP threshold of 40,000 USD/QALY. When considering volume-based procurement, Beijing medical insurance, and Heilongjiang province medical insurance (covering the costs of encorafenib, binimetinib, and cetuximab), triplet therapy was cost-effective compared with the control therapy or doublet therapy as second-line or subsequent treatment for mCRC patients using volume-based procurement combined with Beijing medical insurance at the WTP threshold of Beijing. Doublet therapy was cost-effective compared with the control therapy for mCRC patients using volume-based procurement combined with Heilongjiang medical insurance at the WTP threshold of Heilongjiang province (WTP of Beijing: 95,069.58 USD; WTP of Heilongjiang province: 22,542.51 USD) (Table 4).

## 4 Discussion

In this research, we first applied PSM to evaluate the cost-effectiveness of triplet therapy (encorafenib + binimetinib + cetuximab) compared to doublet therapy (encorafenib + cetuximab) or control group (cetuximab + FOLFIRI) as second-line or subsequent treatment for advanced or mCRC patients with BRAF V600 E mutation in China. Based on the NCCN guideline (colon cancer), for the advanced or mCRC patients with BRAF V600 E mutation positive, the second-line and subsequent therapy option was encorafenib +(cetuximab or panitumumab) or encorafenib+ (cetuximab or panitumumab) + FOLFOX (NCCN, 2025). As the cost-effectiveness evaluation of these treatment strategies was not clear in China, we conducted the cost-effectiveness analysis of triplet therapy compared to doublet therapy or cetuximab plus FOLFIRI treatment for advanced or mCRC patients with BRAF V600 E mutation, providing a reference for clinical doctors, policymakers, and patients in clinical practice. Through searching several public databases, there was only one economic study evaluating the cost-effectiveness of encorafenib, binimetinib, and cetuximab in BRAF V600 E-mutated mCRC in the United States (Li et al., 2021). Although the clinical trial chose the strategy of cetuximab and irinotecan or cetuximab and FOLFIRI as the control group, the Chinese CACA guidelines for holistic integrative management of colon cancer (2024 Edition) and Chinese protocol of diagnosis and treatment of colorectal cancer of the National Health Commission (2025 edition) recommended the BRAF inhibitors combined with anti-EGFR agents as treatment options for mCRC patients with BRAF V600 E mutation positive (Chinese Anti-Cancer Association, 2025; Jin et al., 2026), which was more like the doublet therapy in the clinical trial (BEACON). Therefore, we were more inclined to compare the cost-effectiveness of the triplet therapy with that of the doublet therapy in this study. We conducted the cost-effectiveness of triplet therapy from the perspective of the Chinese payer, and we obtained results similar to the previous study, showing that triplet therapy and doublet therapy were not cost-effective regimens for patients with pre-treated mCRC with BRAF V600 E mutation.

Based on our sensitivity analysis, the results indicated that the factor of significant impact on the ICER of triplet therapy vs. doublet therapy was the cost of binimetinib. The significant factors influencing the ICER of triplet therapy vs. the control group were the cost of encorafenib and the cost of binimetinib. The factor influencing the ICER of doublet therapy vs. the control group was the cost of encorafenib. These results indicated that the main factor affecting the cost-effectiveness of different treatments was the price of the targeted medicine. With the WTP threshold of 40,000 USD/QALY and the cost for encorafenib, binimetinib, and cetuximab fluctuating within 20% of the base value, triplet therapy or doublet therapy proved to be not cost-effective in comparison to cetuximab plus FOLFIRI. This study performed a routine safety assessment to determine adverse event severity grades, adhering to the National Cancer Institute Common Terminology Criteria for Adverse Events (version 4.0) (National Cancer Institute, 2023). All SAEs were presumed to occur during the initial treatment cycle, and their costs were calculated by multiplying the specific adverse reaction management price by the occurrence rate (Su et al., 2021). According to the survival curve data from the clinical trial, the simulation time in our study was 10 years, which could reflect 99.3% of the patients’ lifetime. To investigate the influence of simulation time, a subgroup analysis was conducted with a simulation time of 5 years, which could reflect the lifetime of about 98% of patients. Based on our subgroup analysis results, it could be observed that as the simulation time shortened, triplet therapy or doublet therapy was still not cost-effective compared to cetuximab plus FOLFIRI as second-line or subsequent treatment for advanced or mCRC patients with BRAF V600 E mutation-positive status (Table 4).

To explore the structural uncertainty related to long-term extrapolation, we applied the Gamma model, Log-Normal model, and Weibull model, which had lower AIC values and BIC values to simulate the survival of mCRC patients. It indicated that no matter which model was applied, triplet therapy was still not cost-effective compared with the control therapy or doublet therapy as second-line or subsequent treatment for mCRC patients with a BRAF V600 E mutation at a WTP threshold of 40,000 USD/QALY. At present, China’s medical institutions could reduce drug prices through volume-based procurement. According to statistics, the average drug price dropped by about 58%. At the same time, with the medical insurance of patients, the drug price would further decline. At the same time, patients would further reduce drug prices through their own medical insurance. Besides, different regions in China had different rates of medical insurance reimbursement. In economically developed regions, such as Beijing, the proportion of medical insurance reimbursement was about 85%, and in economically underdeveloped regions, such as Heilongjiang province, the proportion of medical insurance reimbursement was about 75%. The *per capita* GDP of different regions was also different, which indicated the WTP would vary among patients from different regions of China. In conclusion, drug prices would be further reduced by different types of medical insurance on the basis of volume-based procurement in the future. Our study also selected patients from Beijing and Heilongjiang province separately to conduct the cost-effectiveness evaluation for scenario analysis. Based on the *per capita* GDP of Beijing and Heilongjiang province, we used three times the *per capita* GDP as the WTP threshold to conduct the sensitivity analysis (Beijing: 95,069.58 USD; Heilongjiang province: 22,542.51 USD). In our study, we found triplet therapy was cost-effective compared with the control therapy or doublet therapy as second-line or subsequent treatment for mCRC patients using volume-based procurement combined with Beijing medical insurance at the WTP threshold of Beijing. Doublet therapy was cost-effective compared with the control therapy for mCRC patients using volume-based procurement combined with Heilongjiang medical insurance at the WTP threshold of Heilongjiang province.

Our study had certain limitations. Firstly, as Binimetinib did not enter the Chinese market, the price of the Lao generic drug of Binimetinib was provided by the overseas drug purchase platform website (https://www.zhaoyaow.com/bmtn/jg/14380.html), through which patients could obtain overseas drugs (Captain Medicine). With the implementation of medical insurance negotiations for the introduction of the targeted drugs, the prices of the targeted drugs might decrease. Secondly, the utility values for health status were archived from prior literature; health states of the advanced or mCRC patients with BRAF V600 E mutation positive using triplet therapy or doublet-therapy, or cetuximab plus FOLFIRI were not explicit. Since the clinical trial we referred to was not favorable to the quality-of-life evaluation on patients, this study obtained relevant quality-of-life scores of the patients through searching other pertinent literature, which could indirectly reflect the health utility of the targeted patients. Thirdly, only the prices of SAEs were taken into consideration for this study, with the incidence rate surpassing 5% and the adverse reaction grading level exceeding or equal to 3. Although other adverse reactions might influence the results, the sensitivity analysis suggested that adverse reactions had a relatively minor impact on the robustness of the ICER.

## 5 Conclusion

Our results suggested that triplet therapy was not cost-effective compared to doublet therapy and control therapy as second-line or subsequent treatment for advanced or mCRC patients with BRAF V600 E mutation at a WTP threshold of 40,000 USD/QALY.

## Data Availability

The datasets presented in this article are not readily available because Based on a clinical trial which has been published. Requests to access the datasets should be directed to Tong Liu, 332581817@qq.com.
